# The mysterious sense of smell: evolution, historical perspectives, and neurological disorders

**DOI:** 10.3389/fnhum.2025.1588935

**Published:** 2025-06-13

**Authors:** Thomas Brandt, Doreen Huppert

**Affiliations:** ^1^German Center for Vertigo and Balance Disorders, Klinikum der Ludwig-Maximilians-Universität München, Munich, Germany; ^2^Department of Neurology, Klinikum der Ludwig-Maximilians-Universität München, Munich, Germany

**Keywords:** smell, fragrances, perfumes, olfaction, evolution of olfaction, imaging disorders of olfaction, history of fragrances and perfumes, neurology of olfaction

## Abstract

Phylogenetically, the chemical sense of smell is the oldest of all sensory modalities in terrestrial and aquatic organisms. For most non-human species in the wild, it is essential like other senses for survival because it aids nutrition, detection of environmental threats, and mating. In contrast to other senses, olfaction holds some unique properties: vertebrates, humans, and other mammals can distinguish many thousands of different odors due to genetically determined specific odorant receptors which have a lifespan of about 1 month and then are continuously replaced by neuroneogenesis in the olfactory epithelium. From a historical perspective, fragrances and perfumes played a significant role in the most influential ancient cultures, Egypt, Greece, and China. Most important was the belief in the magic power of fragrances—which were classified as “pleasant” or “unpleasant”—for medical treatment, religious or funeral rituals, e.g., preparing the bodies of the deceased for the assumed life after death, purification and divine favor. Further perfumes were used to cover natural body odor, for personal grooming, or to offer a potential hedonic odor in sexual selection. In contemporary medicine, the potential diagnostic value of olfactory loss as a biological marker for an impending neurodegenerative disorder such as Mild Cognitive Impairment, Alzheimer’s disease, Parkinson’s disease, or estimating the inflammatory activity in Multiple Sclerosis is increasingly recognized. The regeneration of odorant receptors and inhibitory interneurons provide the basis for long-term recovery of loss of olfaction due to respiratory infections, for example in pandemics like COVID-19 or after a head trauma. Imaging disorders of olfaction disclosed clinically relevant structural changes of the brain network of olfaction and the intimate reciprocal interaction with other networks to subserve higher cortical functions such as an impressive specific odor memory, quality of life, emotion, cognition, selection of food, social interaction, stress, and depression. The latter higher olfactory functions often remain undetected by both patients and their doctors. A more intensive implementation of olfactory function and clinical testing should be considered in medical training.

## 1 Introduction

From an evolutionary perspective, smell is the oldest sense, as experimentally shown by Nijland and Burgess (2010) who found a behavioral response to odorant molecules sensed by airborne volatile metabolites in *Bacillus licheniformis*. However, one should distinguish between so-called micro-and macrosomatic species. Bacteria, single-celled microorganisms, belong to the group of prokaryotes without cognitive functions. In later phylogenesis, it is remarkable that the sense of smell comparatively shows little development and alterations. In most non-human species, olfaction is essential for survival for three major functions: (1) nutrition (foraging and testing edibility), (2) detection of serious threats that may go undetected by other senses (avoidance of environmental danger), (3) mating (finding the right partner for reproductive purposes).

In humans and other mammals, the sense of smell, in contrast to other senses, holds unique structural and functional properties:

- Mammals and humans can distinguish many thousands of different odors, the majority of which cannot be identified by name, but others can be recognized for life after a few exposures in childhood, characteristic of a precise often emotionally linked smell memory.
- The ability to distinguish many thousands of odor qualities is based on similar specific odorant receptors, the molecular structure of which was first disclosed by the Nobel laureates Buck and Axel (1991). They discovered a large gene family, comprised of some 1,000 different genes, equivalent to the olfactory receptor types (Press Release, 2024).
- The huge number of different smell receptors stands in an unexplained contrast to the cooperative sense of taste with only six receptors for the flavors of sweet, sour, bitter, salty, umami, and licorice (Brandt et al., 2024).
- The mean lifespan of olfactory receptor neurons is about 1 month with a replacement by mitotic generation from basal stem cells in the olfactory epithelium, an example of an adult neurogenesis over the entire lifespan in animals (Lledo and Valley, 2016) and humans (Glezer and Malnic, 2019; Franco et al., 2025). This recovery is beneficial for the frequent loss of olfactory function in infectious diseases such as COVID-19 (Li et al., 2024).
- A loss of olfactory function may be an early indicator of an impending neurodegenerative disease such as Parkinson’s disease or Alzheimer’s disease (Marin et al., 2018).
- The central network of the olfactory system must be extensive to subserve higher cortical functions such as quality of and satisfaction with life, emotion, cognition, social interactions, dietary choices, stress, and depressive symptoms (Bratman et al., 2024).
- When other sensory systems, especially vision, are impaired, the sense of smell takes on a particularly important compensatory role. Odor thresholds and discrimination of dangerous decomposing food have been shown to be superior in blind subjects to those of controls (Çomoglu et al., 2015; Guducu et al., 2016; Kolarik and Moore, 2024).

In the following review, we will focus on some aspects of the human sense of smell whose importance is often underestimated in today’s society, although well recognized in the animal world and easy to observe in the behavior of our pets. The review includes facets of phylogenetic evolution, antique sources on functions and applications of fragrance in various cultures, the development and usage of perfumes, and the neurology of smell disorders.

## 2 Phylogenetic evolution of olfaction

Phylogenetic evolution refers to the development of biodiversity and how different organisms from bacteria, worms, and insects to vertebrates, mammals, and primates are related and have evolved from common ancestors over time. The research findings are extensive and involve molecular, genetical, biological, or clinical disciplines. Starting from a genetic radiation in reptiles 200 million years ago, terrestrial vertebrates can differentiate millions of odorants and in the mammalian genome the olfactory receptor gene family is the largest, comprising 1% of all genes (Hoover, 2010). Amniotes originated on land but some became aquatic and therefore had to adapt their sense of smell to underwater olfaction which created amphibious species with both capabilities, aquatic amniotes (Kishida, 2021). The separation of two main chemical senses, olfaction and gustation, remained largely preserved and independent of the aquatic habitat in which most ancestral and numerous vertebrates, fish, live (Derby and Caprio, 2024). With a view to neuroethological principles, the study of insect olfaction revealed how the ecological environment and other selective pressures forced the fascinating diversity of this large group of species (Hansson and Stensmyr, 2011). On the contrary, the general features of the olfactory system are remarkably consistent across vertebrates, e.g., with respect to the vomeronasal system (Eisthen, 1997). The nasal cavity, which serves olfaction and respiratory air-conditioning, shows a development with anatomical reduction of the vomeronasal system in both cetaceans and primates (Van Valkenburgh et al., 2014). A correlated transformation affected olfaction, mastication, head movements, and ventilation as derived from mammalian fossils and called ortho-retronasal olfaction (Rowe and Shepherd, 2016). As summarized by Ache and Young (2005), “There are striking similarities between species in the organization of the olfactory pathway, from the nature of the odorant receptor proteins, to perireceptor processes, to the organization of the olfactory CNS, through odor-guided behavior and memory.” Finally, the frequently heard opinion that humans have a poorer sense of smell than other mammals is contradicted by some scientists who even emphasize that humans can discriminate a similar range of odors and are more sensitive to some odors than rodents or dogs (Mc Gann, 2017).

## 3 Smell in antiquity

### 3.1 Ancient categorization of different smells in Greek antiquity

Ancient natural philosophy also included attempts to systematically name odors and their associated emotional components. Different positions (Wöhrle, 1987) have been taken: Plato (427–347 BC) mentions in ‘Timaeus’ that only “the pleasant” and “the unpleasant” odors can be individually distinguished without further specification. His concept was based on the assumption that the elements themselves are odorless and that smell arises from certain transformation processes that occur in the transition from water to air and from air to water as smoke or mist. Aristotle (384–322 BC) takes a somewhat different view, especially in his ‘De Anima’ and ‘De *Sensu*’. He writes that smelling takes place through a medium, air or water. The air inhaled through the nose and the pneuma that flows in from the heart via the brain vessels mix in the ‘poroi’, the canal-like pathways between the nose and the brain. In ‘De *Sensu*’ he writes that the olfactory organ is located in the brain (Jütte, 2000). He described the difficulty in determining the properties of odors because of the limited competence of the human sense of smell, which therefore only allows a rough emotional distinction. According to Aristotle, smell and taste seem to behave analogously, with the types of taste corresponding to those of smell. He assumed specific odors that can only be named accordingly if they are parallel to taste, such as a sweet, sharp, tart, pungent, oily, or sour odor. Both authors attribute the accompanying affects to the biological needs of humans. An odor is perceived as pleasant if it restores the natural state of the body or if it is beneficial to this state (Wöhrle, 1987; Jütte, 2000; Jütte, 2012). Theophrastus (371–287 BC), a student of Aristotle, notes that there are seven types of smell, although he does not specifically list these odors. He is more cautious about the terminology and emphasizes that one should not speak of a bitter, salty, oily, or sour odor. Galen (ca. 129–216 AD) also divided odors into those that smell good and those that smell bad, stating that unpleasant odors differ from each other not considerably. In contrast to Aristotle and Theophrastus, he excluded the tart smell (Wöhrle, 1987). In ‘De placitis Hippocraticis et Platonis’ Galen writes: “Incidentally, the organ of smell is not located in the nasal cavities, as most people assume, but in the outermost sections of the anterior ventricles of the brain, where the nasal passages extend. It is in this section that the ventricles contain the most air” (Jütte, 2000). This anatomical conception contributed to the fact that for centuries the physiological process of sensory perception via the nerve stimulus and the transmission of stimuli to perception in the brain was misjudged. The Arab scholar Avicenna alias Ibn Sinai (980–1037 AD) summarized Aristotelian and Galenic knowledge in his doctrine of the senses. He also explained the smelling process by inhaling air that has absorbed the odor from a smelling body. This air then comes into contact with the frontal part of the brain and is detected by the olfactory system. He raised the theory of perception to a new level and brought memory into play. In his work ‘Risala fi n-nafs’ (‘On the Soul’) he writes: “Perception is either external—the five senses—or internal—the common sense, imagination, judgement and memory” (Jütte, 2000). The memory serves to retain the thoughts or concepts with which one has sensually grasped the objects. The power of imagination restores what is blurred in the memory, the power of judgment distinguishes what the imagination considers to be correct or incorrect before it is transferred to the memory (Jütte, 2000). It was not until around 1500 AD that ‘fila olfactoria’ were recognized as olfactory nerves on the basis of cadaver studies (Jütte, 2012).

### 3.2 Historical reports on the relation of olfaction and seasickness

In ancient Greece and Rome, reference is made to the importance of the sense of smell for susceptibility to and treatment of seasickness. In numerous descriptions of seasickness, two factors are repeatedly mentioned: first, the current status of the mental condition and stability; and second, the significance of pleasant or unpleasant odors (Huppert et al., 2016). The unfamiliar odor of the sea and inside the ship causes nausea and vomiting, in contrast to the familiar smell of drinkable river water. Plutarch mentions in ‘Aitiai Physikai’ (‘Questiones naturales’; ‘Natural phenomena’) (Plutarch, 1965) that unpleasant odors induce seasickness in susceptible persons. He writes: “Is it because, among the sensory perceptions, odour and, among the emotions, fear cause the most seasickness? But on the sea they feel aversion to the odour because of its unfamiliarity and are afraid because they distrust the present situation and the future.” ‘Sentina’, the ship’s slop or bilge water is also mentioned as a provoking factor. Caesar writes in ‘Bellum civile’ (Caesar (Caius Julius), 1914) that sentina, the dirty water in the bilge, a foul-smelling broth, the odor of which was constantly noticeable in the lightly built wooden antique ships, caused seasickness among the recruits. In addition, the bilge served as a lavatory in stormy weather and probably also collected the vomit of seasick persons who were not on deck. In ‘Circulio’ Plautus uses ‘sentina’ together with the term ‘nautea’ for indisposition and nausea, which makes an etiological connection between ‘nausea’ and ‘nautea’ likely (Plautus, 1963). Laena, an old drinker, sings the praises of wine: “For the scent of all ointments is nautea, compared to yours, you are myrrh to me, you cinnamon, you rose, you saffron and cassia, you most precious ointment, for where you are poured out, there I would like to be buried.” ‘Nautea’ becomes the cause of ‘nausea’ through the mediation of the sense of smell. The linguistic relationship between the two words proves the close connection between the sense of smell and the development of nausea and vomiting in the consciousness of the ancient world. Juvenal writes in ‘Satura’ about the sickening smell of bilge water (Juvenal, 1928): “… Then the wake (‘sentina’) is nauseating, then the firmament turns (‘tunc summus vertitur aer’).”

Aromatic scents were also used to prevent and treat seasickness. Recommendations were to smell pleasant odors, such as thyme, mint or quince, then to inhale other pleasant scents, such as fennel, rose petals, boiled in wine or mint. Athenaios writes in ‘Deipnosophistae’ (Athenaios, 2012) how Aphrodite sent aromatic scents to seasick people on the ship after they had already given up hope of survival. Other recommendations are to drink wormwood, fast during the sea voyage, or impose a certain diet such as dried or cooked lentils with mint and bread, powdered in fragrant wine. An example of this is the story of the merchant Herostrat and the statue of Aphrodite that he had bought in Cyprus and wanted to take home with him. “When suddenly a storm broke over him as he was approaching Egypt, and it was impossible to tell where in the world they were, they all took refuge with the statue of Aphrodite and begged her to save them. The goddess now—since she was fond of the Naucratites—suddenly saw everything lying beside her, abundantly sprinkled with fresh myrtle, and filled the ship with the most pleasant fragrance, while the travelers had already given up hope of rescue because of the severe seasickness that prevailed and because of all the vomiting. And as the sun shone forth, they saw the anchorages and reached Naukratis.” The miracle that Aphrodite performs here can be seen as a causal therapy using the scent of myrtle. Aemilius Macer in ‘De viribus herbarum’ (Macer, Aemilius (pseudonym), 1932) reports about four remedies for ‘nausea’ (used here synonymously with seasickness), the satureis shrub (‘satureia’), dill (‘anethum’), fennel (‘feniculum’), pennyroyal (‘pulegium’). These plants are particularly strongly scented species, some of which contain essential oils. So in antiquity, therapy and prophylaxis with fragrances appeared to be useful. Further examples are found in Plinius ‘Historiae Naturales’ (Plinius (The Elder), 1951), where he recommends various strongly scented plants such as mallow (‘malva’), rose leaves boiled in wine, and pole mint (‘puleium’) as a therapy against ‘nausea’ (not clearly labelled here as seasickness). Oreibasios in ‘Synopsis ad Eustathium’ (Oreibasios, 1926) also gives therapeutic recommendations against vomiting during sea voyages using various foods by fragrant substances: “… or polei and water with very fine barley flour or small, fragrant wine with water and also very fine flour. But to counteract the unpleasant odours in the ships, you have to smell either quinces from Kedonya or thyme or polei.” So, the dietary suggestions all contain fragrant substances, presumably because the unpleasant odor on board was thought to be partly responsible for the occurrence of seasickness.

Wormwood and mint, which were rubbed into olive oil and fragrant wine vinegar, were also to be applied to the nostrils repeatedly. This was intended to mask the unpleasant odors, especially the unfamiliar smell of salt water and the ‘sentina’. Seneca in ‘Ad Lucilium epistoles’ (Seneca Minor (Seneca the Younger), 1979) anoints himself with fragrant oil to refresh himself: “… As soon as I was in order with my stomach, which, as you know, does not get rid of seasickness as soon as I leave the sea, and had refreshed my body with anointing oil, I began to think about it …”

### 3.3 Meaning of olfaction in ancient Egypt

**Figure 1:**
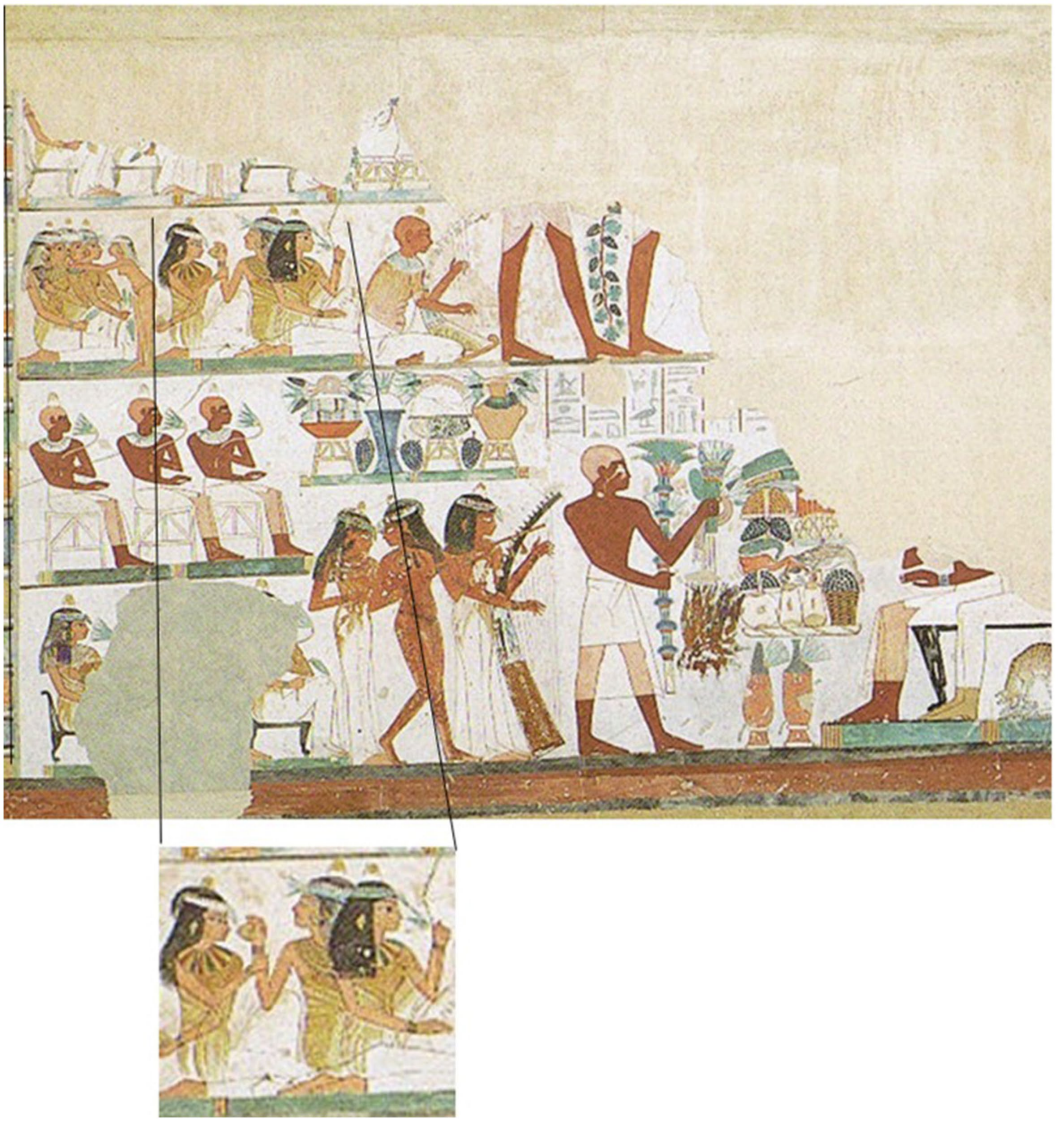
Detail from the depiction of the ‘Beautiful Feast of the Desert Valley’ in the Tomb of Nakht (TT52). It shows three ladies spraying each other with pleasant fragrances. Modern illustration by Norman de Garis Davies, Nina Davies (2-dimensional 1-to-1 Copy of a 15th century BC Picture) in: Matthias Seidel, Abdel Ghaffar Shedid, Das Grab des Nacht. Kunst und Geschichte eines Beamtengrabes der 18. Dynastie in Theben-West. Von Zabern, Mainz 1991.

The general differentiation of odor into ‘good’ and ‘bad’ is a characteristic of many cultures. This was also the case in ancient Egypt. The hieroglyphic script in various written sources (including papyrus scrolls, wall inscriptions, engravings in stone) shows the signs of fish and birds as prototypes of odor in ancient Egypt, and these were signs of a collective olfactory value. There are no references to individual olfactory perceptions in the ancient sources. Fish and birds lived in the natural environment in Egypt, the swamps of the Nile delta in the north of Egypt, and this stank. On the other hand, the city of Theben is described as clean and home to families. It is associated with myrrh, has the odor of perfume and the inhabitants are dressed in fresh garments. The ancient Egyptians believed in two concepts: ma’at und isfet. Ma’at is the world of order and justice, isfet that of chaos and evil. The king embodied ma’at, while the world’s natural state is that of isfet, a state of chaos, evil, lies, injustice and stench. The world smelled naturally bad, isfet smelled like fish and birds. The king’s task was to banish the stench of fish and birds and to establish ma’at. Then the people could go back to their cities, to the sweet odors, as written sources describe. Thus, sweet odors were synonymous with the result of the king’s efforts for society, allowing people to live in peace surrounded by good smells (Goldsmith, 2019). Egyptians also used scents in temples to honor various deities and perform mummification of the Pharaohs. Common fragrance ingredients were incense and myrrh. Ingredients were mainly imported from abroad and were expensive, which is why the best fragrances were allowed to use only the rich up to the king (Goldsmith, 2019). Pictorial grave decorations show scenes in which women smell fragrant bottles or flowers (Figure 1).

The fact that odors have been connoted with geographic areas referring to ma’a’ or isfet could be explained how odor- and context-dependent memory seem to work in the brain. Olfactory memory is one of the strongest types of memory. Olfactory information is processed quickly and is retained longer as compared to other memories. Experiments showed that olfactory memory does not or very slowly declines over time—it is largely the same after 5 min and a year later (Engen, 1982). When olfactory memory is combined with contextual memory (Herz and Engen, 1996) or cross-modal paired-association it is strong and effective (Hamzeloo et al., 2025).

## 4 Fragrances and perfumes

**Figure 2:**
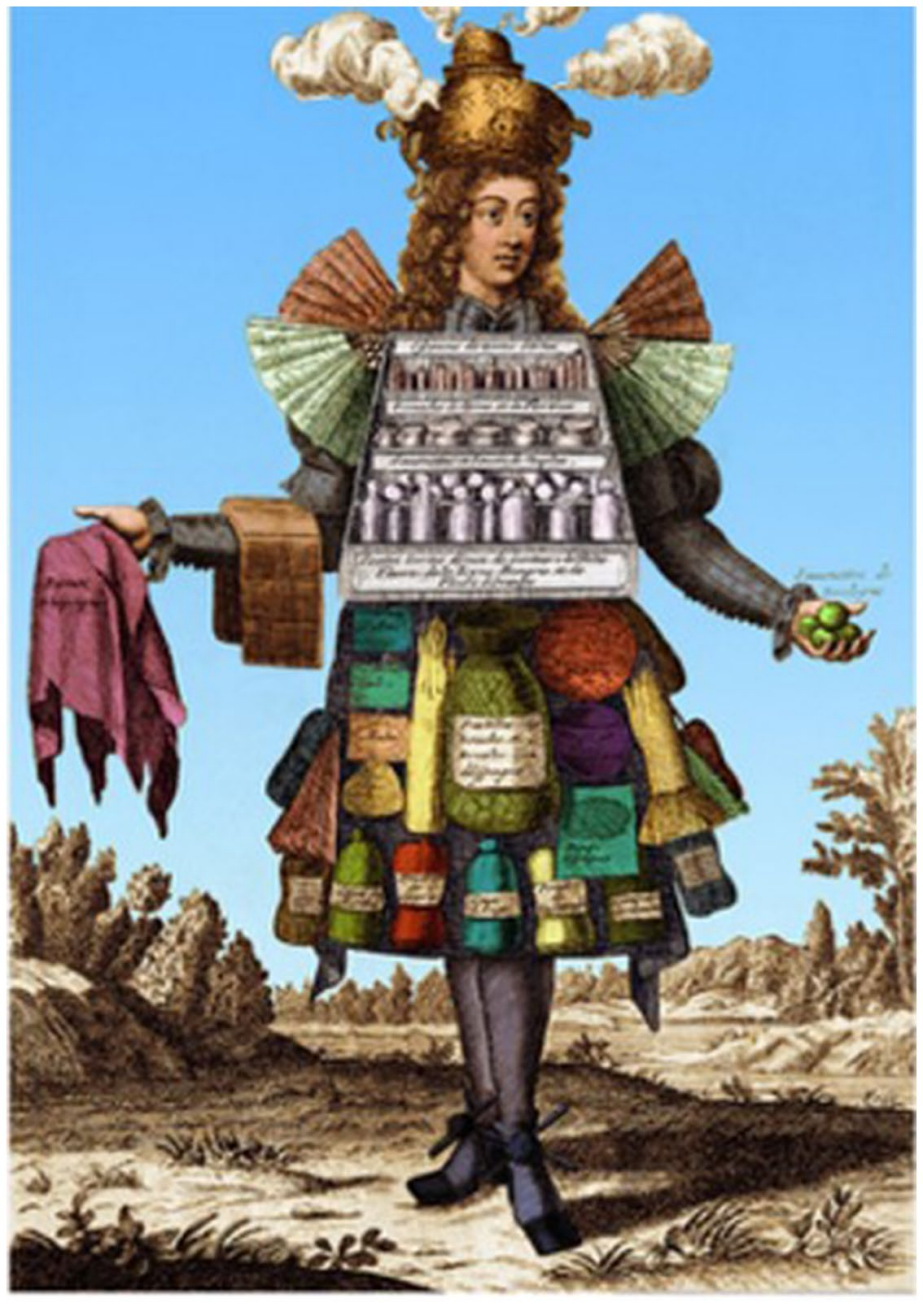
An engraving by Nicolas de Larmessin (1638–1694), a French engraver, depicting a parfumeur in his typical costume.

From antiquity to the present day, perfumes have been used in most cultures to cover individual body odor or to provide a positive, attractive and interesting impact, or a smell that characterizes a certain social group (Capparuccini et al., 2010). In the 17th century, a guild of glove makers and perfumers was founded in France (Figure 2).

The potential hedonic role of olfaction in sexual selection was investigated in a contemporary behavioral study which revealed that non-pheromonal olfactory cues were involved in mate choice with strong hedonic responses that could dominate visual aspects in a cross-modal interaction (Capparuccini et al., 2010). Essential oils, a mixture of natural aromatic volatile oils extracted from plants, were widespread in Egyptian, Greek, Persian, and Chinese cultures and are still common today as complimentary remedies in medicines, aroma and massage therapies and food products (Sattayakhom et al., 2023). Today’s eau de toilette and perfume articles sometimes refer to the ancient roots by naming their products “Egyptian rose” (botanical name: Scabiosa atropurpurae) or “Etruscan water” for perfumes emphasizing a spicy-woody smell. In a review, Sattayakhom et al. (2023) included 70 studies and concluded that essential oils showed beneficial effects including antistress, antianxiety, analgesic, cognitive, and autonomic effects, which led them to recommend it as an alternative therapy. In traditional Chinese medicine, inhalation in the forms of smoke, steam vapor, medicated pillows and aromatic sachets have been widely used for treatment of respiratory diseases since antiquity but only a few aromatic inhalation products have been approved by the China Food and Drug Administration (Miao et al., 2015). The fragrant camphor tree (botanical name: *Cinnamomum camphora*) was used as a fumigant in the era of Black Death (Chen et al., 2013), a pandemic outbreak throughout Europe and Asia that killed half of the population. Camphor has been used in cosmetics, in household cleaners, and as food flavoring with biological properties such as insecticidal, antimicrobial, antiviral, and anticoccidial effects; however, it is quite toxic a substance which significantly limits its use (Chen et al., 2013). Before we focus on the use of fragrances and perfumes as medical substances, some still used in the present day, we will first compile historical sources from different ancient cultures.

### 4.1 The use of fragrances in antiquity

**Figure 3:**
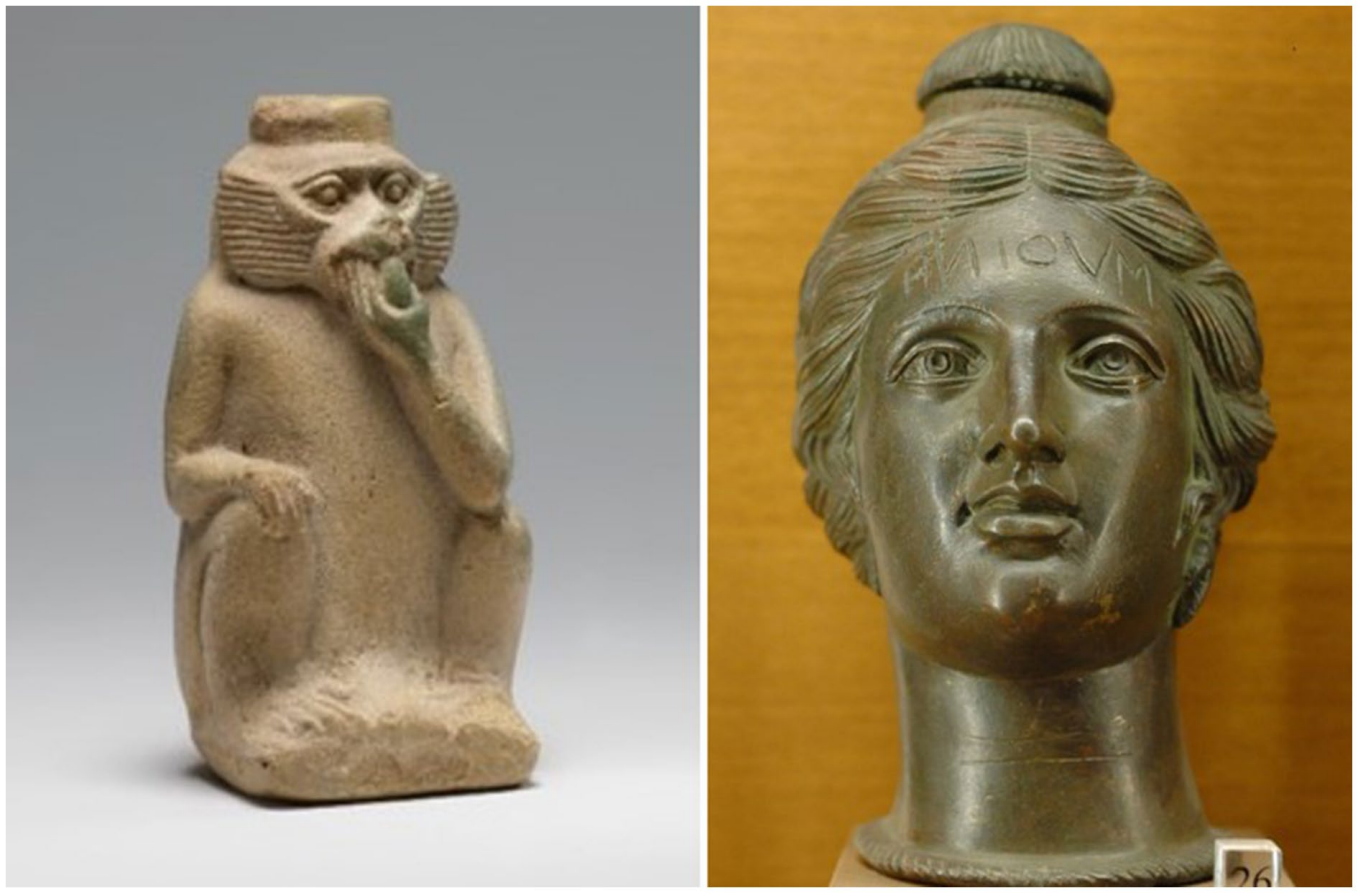
Left: Perfume vessel in the shape of a monkey from the Egyptian period of the New Kingdom, Dynasty 18, ca. 1550–1295 BC. Metropolitan Museum of Art, New York. Right: Etruscan perfume vase shaped like a female head. Inscribed is the word ‘suthina’ (‘for the tomb’) in retrograde Etruscan script. Bronze, early 2nd century BC. Louvre Museum.

Good odors played a major role in ancient Egyptian society. Egypt was famous for its perfume among other ancient civilizations. Perfume was mainly produced in Alexandria, which was also a famous marketplace for several Egyptian products such as myrrh. Mendes, the ancient Egyptian city of Djedet (today Arabic تل الربع Tall al-Rubˁ) in the eastern Nile Delta, was also famous for its perfume. The manufacture of perfumes is depicted in several ancient Egyptian temples. Perfume recipes are inscribed on the walls of the laboratories of the Ptolemaic temples. The manufacturing process of the fragrances consisted of three techniques. The substances were powdered, mixed together and then heated over a fire, several herbs and plants such as iris were used to make perfume. Iris root or balsam was the base for perfumes; the most fragrant oils in ancient Egypt were myrrh, frankincense and lily, which were mixed with other essentials from flowers, fruits and herbs (Fadel, 2020). Various words were used that referred to perfume such as “ndm.st” for pleasant and “rdw” for the jar containing the substance (Fadel, 2020). Different types of perfume, stored in elaborate vessels, as well as fragrant flowers were offered to the deities, which is depicted in the art of ancient Egypt (Byl, 2012). Perfume bottles in the shape of a monkey have survived from the new Kingdom (Figure 3 left).

Large quantities of incense, perfume, and scented flowers were used in the temples to please the deities (Fadel, 2020). The temple ritual of the cult statue was a central element of this and was performed every morning on the principal cult statue in every Egyptian temple, by anointing the cult statues with precious oils and unguents. Especially in the temples of the Ptolemaic period, illustrations show how perfume as well as cloth were offered to various deities. The blue lotus flower was considered the perfume of the sweat of the good Ra. In the temple of Hathor at Dendera, there is a scene in which the king offers the ‘horizon of lotus’ cloth to the goddess Isis which is said to be soaked in lotus scent (Fadel, 2020; Byl, 2012).

Ointments and perfumes were widely used in antiquity either as part of everyday life and personal hygiene or on special occasions such as funeral rites or religious rituals in preparing the bodies of the deceased for the assumed life after death, purification, and divine favor. They were first established in Mesopotamia, further developed in Egypt, and then spread to Greece and the rest of the Mediterranean area. The people of antiquity believed that good odors were associated with deities and had a positive effect on health and well-being, as well as having a positive effect on social contacts. The quality of the perfume and its use frequently described a certain social status. Many ancient Greek and Roman writers such as Theophrastus, Plinius, Hippocrates, or Aristotle provided some details about the ancient odors (Voudouri and Tesseromatis, 2015). A connection between divine power and odor can be found in the earliest literature. Good-smelling substances such as myrrh, cassia, and saffron were already mentioned in the Iliad (around 700 BC). Frankincense became established in rituals at the end of the archaic period. Incense was probably brought westwards via Cyprus and burnt in the temple of Aphrodite, for example (Potter, 1999). The association of odor with divine power in the Hellenic world is also found in other states in the rest of the Mediterranean area and the Middle East. However, the choice of specific odors and substances for characteristic sacred acts differed among cultures.

In the Roman world, attitudes toward aromatic scents were very ambivalent. Pliny, for example, had little good to say about them, writing that perfumes were the most superfluous form of luxury and that the early Romans managed without these substances. He describes, for example, how the ruler Caligula sprinkled the walls of his bathhouses or bathtubs with scents. Perfume was considered a luxury, representing moral decay, and even associated with state problems. However, these views on the use of perfume in Rome had no significant influence in real life. Evidence that men used perfume, which was considered feminine, is found in the social elite at that time (Potter, 1999).

The ancient civilization of the Etruscans in Italy between the 9th and 1st centuries BC, i.e., before the Roman Empire, had a tradition of personal grooming and religious rituals, in which perfumes also played an important role (Barker and Rasmussen, 2000; MacIntosh Turfa, 2013). This is reflected by iconographic representations on ceramics and frescoes. Perfume vessels (Figure 3 right), especially alabastrons, aryballoi, and amphorae were either imported from the Eastern Mediterranean or later locally produced to store scented oils and perfumes from the natural ingredients, myrrh, saffron, or resin. Etruscans, obviously inspired by Greek customs, used private perfume vessels as a status symbol of an elite social life, which held their unguents and perfumes. Favorite vessels were inspired by nature and took the form of birds, such as cocks and ducks. These vessels, found in tombs, indicate that the Etruscans were as fond of the luxury of unguents and perfumes as were the Romans in later times (Norton, 1881).

### 4.2 Herbs and diseases in the middle ages

Despite all this use of good odors, there is no doubt that the urban air of the Roman Empire stank from the burning of meat and other food smells, waste, and dead bodies. This was also the case in the Middle Ages—medieval cities stank and foul odors were associated with diseases such as the plague (Jørgensen, 2013). In the Middle Ages, herbs, and certainly fragrant or essential herbs, were used as remedies, and most monasteries developed herb gardens to produce these herbal remedies. The success of these remedies was attributed to their effect on the humors—medieval medicine was based on the theory of humoral pathology. It was said that each person has four humors to which fluids, the black bile, yellow bile, phlegm and blood, were assigned. The balance was decisive for a person’s health; an imbalance of bodily fluids leads to illness. Hildegard von Bingen (1098–1179) a wise abbess, learnt this doctrine developed by Hippocrates; for her, illnesses were embedded in a cosmic context, God and the devil played a role, and demons also brought plagues and death. She wrote texts on natural history and medicine, which we know today from two works, ‘Physika’ (natural history) and ‘Causa et cura’ (medicine). In these works, she discusses the potential benefits of each ailment or herbal remedy, assigning their healing effects to different organs (Bizzarri, 2018). Herbal books from the Middle Ages contain long lists of indications for each plant, most of which are no longer approved today and often even lack the current indication. In this respect, these traditional indications should be used with caution (Uehleke et al., 2012). Fragrancies have been used as a medicine for patients with dementia and behavioral problems, for example, but it has been shown that this therapy cannot change the behavior of patients with dementia (Gray and Clair, 2002). Another application of fragrancies is described for migraines. Here, the use of aromatic oils such as lavender oil, peppermint oil, or rose oil was tested. These oils were said to work through mechanisms that act on vasodilation, inhibition of neurogenic inflammation, and reduction of central pain sensitization, interpreted as a possible non-invasive alternative treatment that can be used additively (Abo-Elghiet et al., 2025).

## 5 Neurology of smell disorders

Olfactory dysfunctions are certainly among the most clinically overlooked sensory symptoms, although impairment of the sense of smell leads to a reduced quality of life. There are two reasons why they may be undetected in the clinical examination: on the one hand, slowly developing minor disturbances may not be noticed by the patients; on the other hand, clinicians often judge the function based on the patient’s self-assessment rather than applying a time-consuming smell test. The most commonly used tests in clinical and scientific studies are (Devere, 2012) the University of Pennsylvania Smell Identification Test (UPSIT) and the Sniffin’ Sticks Test (SST) or its shorter version SST-12 for screening purposes (Schepens et al., 2024).

In case of a pronounced reduction of odor function, the main complaints are impaired ability to perceive and recognize smoke or spoiled foods, distinguish pleasant scents including pheromones, and overcome taste disorders associated with decreased appetite. This can lead to unexplained weight loss and depression (Devere, 2012). Current tests of the sense of smell such as that established by Henkin therefore include detection, recognition, thresholds, magnitude estimation, and hedonic ratings as parameters (Hernandez et al., 2024). A so-called microbiome-gut-brain axis has been described in animals for regulation of the hippocampal serotonergic system for maintenance of homeostasis (Clarke et al., 2013). This “gut-brain axis” seems to control dietary decisions by collaboration of homeostatic and hedonic processes including multisensory input of smell, taste, and sight for regulation of appetite, satiety, and eating behavior (Clarke et al., 2024). Olfaction plays a key role in food appreciation and selection as well as in obesity with brown adipose tissue thermogenesis and substrate utilization (López et al., 2023).

Since the many terms used to describe olfactory dysfunctions such as hyposmia (reduced sense of smell), anosmia (loss of smell), hyperosmia (heightened sense of smell), dysosmia (altered sense of smell) or parosmia and phantosmia are currently not uniformly defined. A group of experts (Hernandez et al., 2023) proposed a standardization of the various terms used in clinical and scientific settings. Parosmia, for example, is an often unpleasant distorted quality of smell due to a mismatch between the memory and the actual experience elicited by a specific odor stimulus (Xu et al., 2025), possibly caused by an inadequate regeneration of olfactory neuronal processing. Phantosmia means a hallucination as sensory perception without an odor stimulus as the trigger.

The clinical relevance of the sense of smell is increasingly recognized. Five conditions may serve as examples:

1. A long-term follow-up study by London et al. (2008) revealed that about 50% of the patients with an initial total smell loss after a head trauma exhibited mild to moderate or complete improvement. Regeneration is based on the continuous replacement of damaged odorant receptors from stem cells in the epithelium’s basal region (Glezer and Malnic, 2019). This process is complex and probably also involves the activity of local inhibitory interneurons (Lledo and Valley, 2016). In central lesions, gene expression profiles of neuroblasts’ migration in the peri-injured cortex have been found (Miyamoto et al., 2025).
2. Olfactory dysfunctions have been described as a marker of the inflammatory activity and progression in MS (Bsteh et al., 2019); a meta-analysis of a total of 1,099 MS cases found a pooled prevalence of olfactory dysfunction in 27.2% (Mirmosayyeb et al., 2022).
3. Olfactory dysfunction is a clinical marker for early stages, disease progression, and cognitive decline in neurodegenerative disorders (Marin et al., 2018); in mild cognitive impairment it can predict Alzheimer’s disease at follow up (Devanand et al., 2000) and Parkinson’s disease (Jankovic, 2008; Alonso et al., 2021; Ma et al., 2023). In Parkinson’s disease multiple sensory disturbances of vision, hearing, smell, taste, and touch occur which affect the patient’s quality of life beyond the motor disorder (Ma et al., 2023).
4. More than 4,000 patients suffering from COVID-19 who were assessed by an international questionnaire self-reported the quantity and quality of three distinct chemosensory modalities (smell, taste, and chemesthesis); the results showed that the impairment was not limited to smell but also affected taste and chemesthesis (Parma et al., 2020). Chemesthesis is not a sensory system on its own but reflects an inadequate stimulation of preferably trigeminal sensors such as the coolness of menthol in mouthwashes or the burning sensation watering eyes when cutting onions; the diagnostic accuracy of screening Sniffin’ Sticks Tests (SST-12) is slightly lower as compared to the validated longer version (Schepens et al., 2024). The pathogenesis of COVID-19 anosmia may be caused by inflammation of the olfactory clefts and damage to the epithelium or the central nervous network (Meng and Pan, 2024). Post-acute central and peripheral sequela of the infection are complex and still not fully understood (Saxena and Mautner, 2024). The presence of parosmia seems to predict persistent quantitative olfactory dysfunction often for years (Gary et al., 2023).
5. Anosmia can sometimes be a key symptom of a congenital condition. The prototypical example of this is the rare Kallmann syndrome, a genetically heterogeneous disorder characterized by the combination of hypogonadotropic hypogonadism with delayed puberty and diminished or absent sense of smell (Fechner et al., 2008; Stamou and Georgopoulos, 2018; Yadav et al., 2025). Congenital ciliopathies can also present with various clinical features and anosmia (Green and Mykytyn, 2010). Brain structural characteristics in congenital and acquired anosmia are altered differently (Manan et al., 2022).

Olfactory training may be an effective and affordable option in the treatment of olfactory dysfunction in patients with head trauma or infections of the respiratory tract but requires adherence to the prescribed training regimen (Li et al., 2024). However, olfactory training should not be seen as a universal therapy for anosmia, given the variability of results (Moura et al., 2025).

## 6 Imaging the central network of olfaction and its disorders

Our sense of smell not only distinguishes countless pleasant and unpleasant fragrance qualities by comparing them with the memory of previous smells, but also simultaneously influences quality of life, emotions, cognition, social interactions, dietary choices, stress, anxiety, and depression (Bratman et al., 2024). An appetizing smell triggers saliva secretion, a foul unpleasant odor, on the other hand, triggers nausea and vomiting. These different odor-dependent behaviors require an extensive central network of olfaction with structural and functional reciprocal interconnections between all sensory systems, memory, the limbic system, attention, and spatial orientation to name just a few. The olfactory-auditory sensory convergence in the basal forebrain of mice may serve as an example. *In vivo* electrophysiological recordings from the olfactory tubercle revealed single units which responded to odors and auditory tones with cross-modal modulation (Wesson and Wilson, 2010). This multimodal convergence allows assignment and identification of two different sensory inputs from a related source at an early stage of odor processing (Wesson and Wilson, 2010), e.g., anticipating the actions of others and, thereby shaping social interactions (Aglioti and Pazzaglia, 2011).

**Figure 4:**
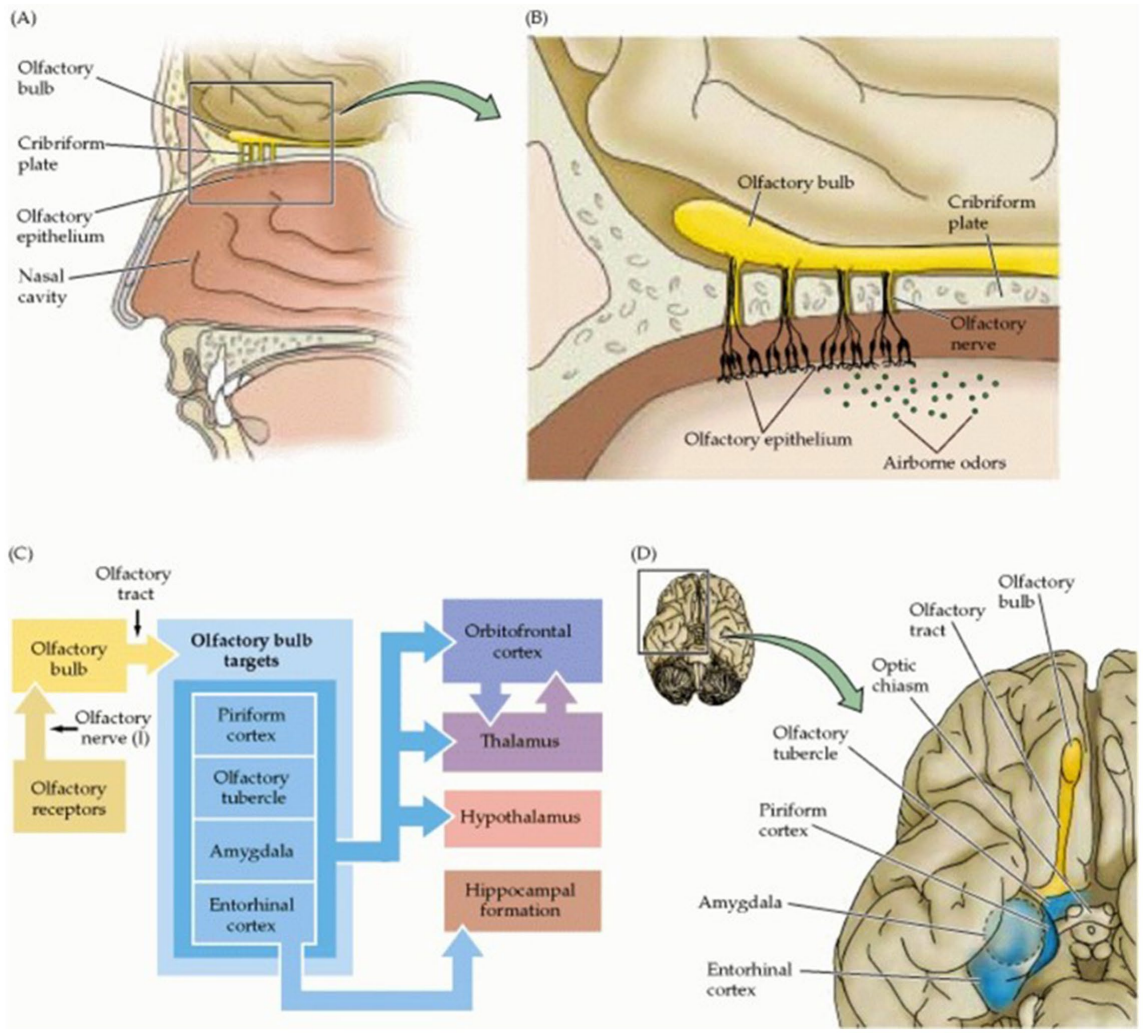
Organization of the human olfactory system. **(A)** Peripheral and central components of the olfactory pathway. **(B)** Enlargement of region boxed in **(A)** showing the relationship between the olfactory epithelium, containing the olfactory receptor neurons, and the olfactory bulb (the central target of olfactory receptor neurons). **(C)** Diagram of the basic pathways for processing olfactory information. **(D)** Central components of the olfactory system. In: Purves D, Augustine GJ, Fitzpatrick D et al. (eds.). Neuroscience. 2nd edition. The Organization of the Olfactory System. Sunderland (MA): Sinauer Associates; 2001. Copyright © 2001, Sinauer Associates, Inc.

The central network of olfaction is diverse and complex due to its intimate integration in various higher cortical functions. The olfactory bulb projects via the lateral olfactory tract to the anterior olfactory nucleus, the olfactory tubercle, amygdala, piriform and entorhinal cortex. The anterior commissure connects the bilateral anterior olfactory nuclei; the piriform cortex is involved in the categorization of odors and has projections to the thalamic complex, hippocampus, amygdala, and orbitofrontal cortex which is supposed to consciously perceive the particular odor. Emotional and autonomic responses are assigned to connections between the entorhinal cortex and the amygdala; the hippocampal complex and the limbic system are involved in the remarkable long-term-memories and associated emotions. Olfaction is still the least understood sensory modality and its central organization in networks is less precise than that of other sensory systems (Figure 4) (Purves et al., 2001). Since the olfactory networks consist of peripheral sinonasal and central neuronal pathways CT scans are paramount for detecting peripheral bony causes while MRI allows for a detailed assessment of the soft tissue of both the peripheral and central components of the olfactory structures and thus enables the detection and localization of the pathological causes (Lie et al., 2021).

Numerous imaging studies using EEG, MRT, or single-neuron representations of odors have disclosed these anatomic and functional task-dependent olfactory functions and disorders of smell (Olofsson and Freiherr, 2019). Intracranial EEG has been used to investigate context-dependent timing and sequence of neuronal oscillations in different structures of the olfactory system (Mignot et al., 2024). Using a similar Intracranial EEG technique, volitional respiratory maneuvers such as active sniffing and apnea were compared (Granget et al., 2025). Preparation of sniff maneuvers and short apnea involved low-frequency bands in the posterior insula and temporal regions extending to amygdala. In MRI studies, functional connectivity showed that sensory cortices converge to central hubs in the association cortices, e.g., the default-mode network. Menelaou et al. (2024) found evidence for two distinct pathways: one from the anterior olfactory nucleus and olfactory tubercle to the orbitofrontal cortex for processing reward, the other from the piriform cortex involving the anterior insula, intermediate frontal sulcus, and parietal operculum. In another fMRI study, lemon fragrance inhalation caused higher levels of alertness in healthy participants which was associated with increased global functional connectivity in the thalamus but decreased activity in other regions (Martial et al., 2023). Graph theory analysis revealed an increased network integration of olfaction and emotion. With fMRI it was also possible to visualize prediction errors of listening to spoken words which were not matched by the expected odor, a paradigm which is mediated by the anterior cingulate cortex (Pierzchajlo et al., 2024). Another fMRI study addressed the question of the differential neuronal basis of lower-and higher-order olfactory functions such as passive smelling, odor encoding, and in particular odor recognition memory (Eek et al., 2023). Successful recognition of familiar odors was associated with neural suppression of regions including the anterior insula, posterior cingulate gyrus, dentate and middle frontal gyrus, amygdala, and piriform cortex; the hippocampus and posterior cingulate were involved in a postrecognition process. Odors also affect the working memory by an inverse correlation between regions associated with olfaction and working memory (Heidari et al., 2024). The latter finding may be relevant with respect to animal experimental behavioral studies which showed that exposure to chemosignals produced by stressed mice were sufficient to impair memory retrieval in unstressed male mice (Gómez-Sotres et al., 2024). This was interpretated as bulb astrocytes providing a link between social odors and the corresponding behavioral performance. New methods allow recordings of single-neuron activity in the piriform cortex and temporal lobe in awake humans performing odor rating and identification tasks (Kehl et al., 2024). This disclosed that piriform neurons preferably encode chemical odor identity and hippocampal activity reflects odor perception, supporting a multimodal role of the human piriform cortex.

Imaging will play a more relevant clinical role in diagnostics of disorders of smell in the future. Although patients with idiopathic olfactory dysfunction in a systematic review uncommonly exhibited MRI structural pathologies such as olfactory meningiomas or neuroblastomas, patients with mixed etiologies showed reduced olfactory bulb and gray matter volumes (Hura et al., 2022). A comparison of olfactory-related brain tissue changes in patients with congenital versus acquired anosmia revealed that acquired olfactory loss led to reduced volumes and thickness of the gyrus rectus, medial orbitofrontal cortex, anterior cingulate cortex, and cerebellum (Manan et al., 2022). Congenital anosmia, however, showed larger volumes and higher thickness in parts of the olfactory network. Temporal lobe epilepsy involves structures of the primary and secondary olfactory cortex such as the piriform and entorhinal cortex, the amygdala and the hippocampus. Accordingly, this condition is characterized by an increased olfactory dysfunction of secondary olfactory structures connected to the limbic system (Schmidt et al., 2024). In patients with paroxysmia, resting state MRI scans exhibited a reduced information flow between memory decision centers, and primary and secondary olfactory areas (Thaploo et al., 2023). In *de novo* Parkinson’s disease, diffusion MRIs at the baseline visit compared with a 48-month follow-up visit revealed structural connectivity changes associated with apathy in patients with dysfunction of smell (Martinez-Nunez et al., 2022). A resting-state fMRI study describes different olfactory network alterations in patients with Parkinson’s disease and mild cognitive impairment (Cieri et al., 2024). On the other hand, in early blind humans increased odor processing performance was found which was associated a significantly higher olfactory bulb volume (Rombaux et al., 2010) and which was associated with an activation of the occipital cortex during odor processing tasks in functional MRI (Renier et al., 2013).

## 7 Concluding remarks

One goal of the selective discourse on the mysterious sense of olfaction was to show that fragrances and perfumes were more significant in the authoritative ancient cultures than in today’s society. In antiquity, the Egyptians, Greeks, Etruscans, Chinese, and Persians believed in the magic power of odors for personal grooming, medical treatment and religious or funeral rituals such as perfuming their dead for the assumed life after death to make the gods friendly. In today’s world smell seems to play a more subordinate role compared to other sensory modalities, although molecular, systemic, and behavioral studies clearly prove how our quality of life, the state of mind, and social interactions still depend on sometimes not consciously perceived memories of smells shaping our behavior. In medicine, the diagnostic importance of olfactory disorders is increasingly recognized as a biological marker. However, the implementation in medical training is very hesitant. The latter statement is hard to believe, but it is supported by clinical studies. An international, cross-sectional survey of current practice in the assessment of olfaction from 18 countries revealed the lowest rates from Japan (1.4%) and the highest from Greece (72.5%). Most UK clinicians do not perform psychophysical smell testing during any of the presented clinical scenarios. Reasons for this clinical deficit were related to service provision such as time and funding limitations (Whitcroft et al., 2024a). There is little standardization of clinical practice and many professionals across specialties were dismissive toward olfactory dysfunction and lacked appropriate knowledge of both its pathophysiology and effects (Whitcroft et al., 2024b). Some of the most important experimental studies in the future will include those of the mechanisms of olfactory receptors in neural regeneration, not only for restoration of smell, but also as a model for central nervous system neuroneogenesis (Franco et al., 2025). In this way, olfactory receptors could serve as potential therapeutic targets to accelerate neuronal repair processes and functional recovery in central nervous system injuries, brain and spinal cord. Another important topic for future research on the olfactory system is the combination of electrophysiological and new imaging techniques such as connectivity studies to elucidate the intimate interaction of the widely distributed central olfactory network with cognition, memory, and emotions. This also involves investigating the dependence of higher order olfactory cognitive functions and the triggering or amplification of depressive disorders (Yuan and Slotnick, 2014).
